# New fossil from mid-Cretaceous Burmese amber confirms monophyly of Liadopsyllidae (Hemiptera: Psylloidea)

**DOI:** 10.1038/s41598-020-74551-6

**Published:** 2020-10-19

**Authors:** Jowita Drohojowska, Jacek Szwedo, Patrick Müller, Daniel Burckhardt

**Affiliations:** 1 Institute of Biology, Biotechnology and Environmental Protection, University of Silesia, 9, Bankowa Street, 40-007 Katowice, Poland; 2 Laboratory of Evolutionary Entomology and Museum of Amber Inclusions, Department of Invertebrate Zoology and Parasitology, University of Gdańsk, 59, Wita Stwosza Street, 80-308 Gdańsk, Poland; 3 Amber Study Group, c/o Geological-Palaeontological Museum of the University of Hamburg, Bundesstraße 55, 20146 Hamburg, Germany; 4 Naturhistorisches Museum, Augustinergasse 2, 4001 Basel, Switzerland

**Keywords:** Entomology, Palaeontology, Taxonomy, Palaeoecology

## Abstract

*Amecephala pusilla* **gen. et sp. nov.** is described and illustrated on the basis of a well-preserved female psyllid (Liadopsyllidae) in a piece of Cretaceous Myanmar amber. The new genus differs from other members of Liadopsyllidae in details of the antennae and forewings. For the first time, the presence of a circumanal ring is documented for Mesozoic psyllids. Based on differences in the length of female terminalia, it is suggested that Liadopsyllidae may have displayed a diversified oviposition biology. As far as known, Liadopsyllidae lack a pulvillus, a putative autapomorphy supporting the monophyly of Liadopsyllidae. An identification key to genera and an annotated checklist of known Liadopsyllidae species are provided. New synonyms and combinations are proposed and the status of the subfamily Miralinae is discussed.

## Introduction

**Figure 1:**
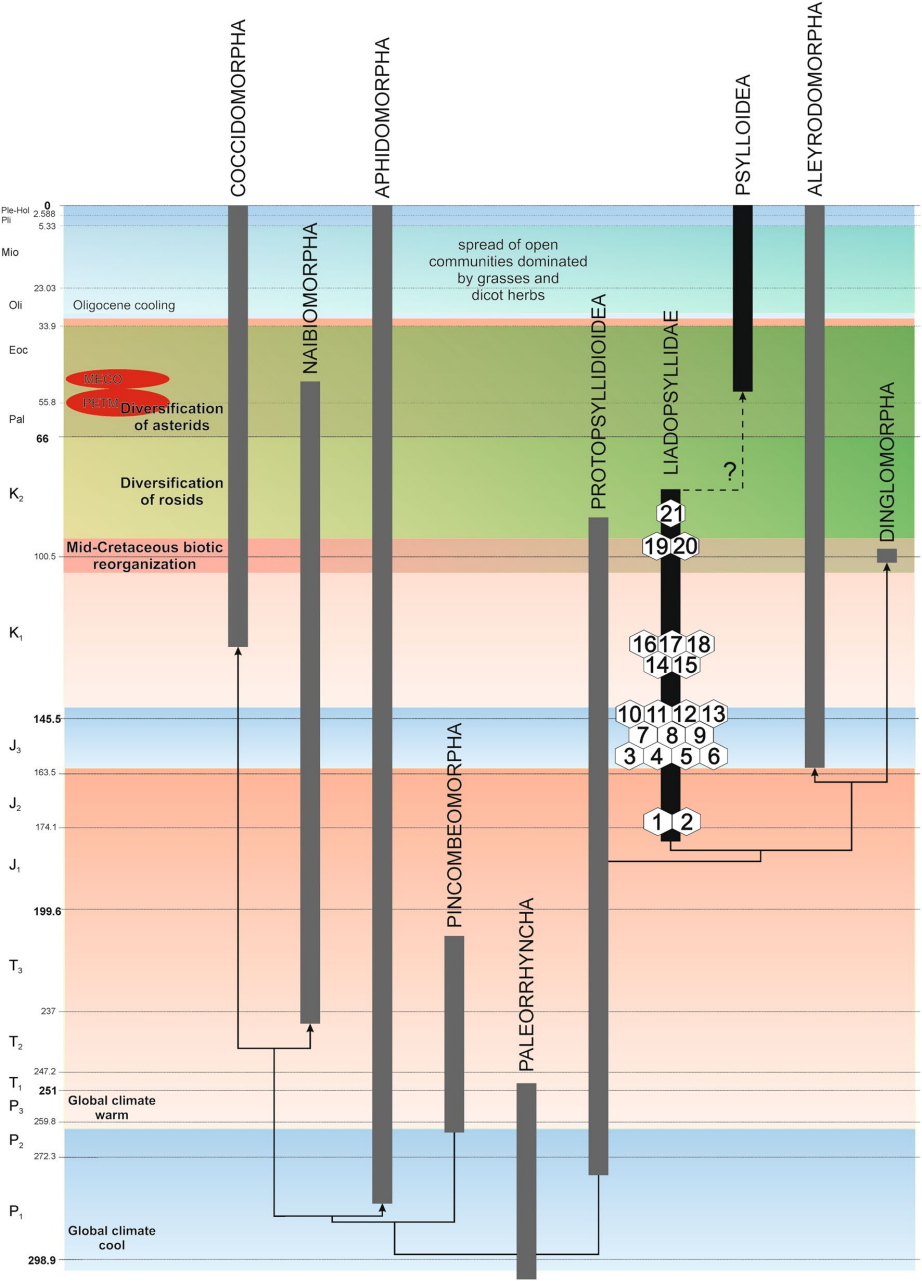
Relationships and stratigraphic distribution of Liadopsyllidae and its subunits within Sternorrhyncha according to Drohojowska & Szwedo^10^, Hakim et al.^11^ and Drohojowska et al.^12^, modified. Numbers denote described taxa of fossil Liadopsyllidae—1: *Liadopsylla geinitzi* Handlirsch, 1925—Lower Jurassic, Mecklenburg, Germany, 2: *Liadopsylla obtusa* Ansorge, 1996—Lower Jurassic, Mecklenburg-Vorpommern, Germany, 3: *Liadopsylla asiatica* Becker-Migdisova, 1985—Upper Jurassic, Karatau, Kazakhstan, 4: *Liadopsylla brevifurcata* Becker-Migdisova, 1985—Upper Jurassic, Karatau, Kazakhstan, 5: *Liadopsylla grandis* Becker-Migdisova, 1985—Upper Jurassic, Karatau, Kazakhstan, 6. *Liadopsylla karatavica* Becker-Migdisova, 1985—Upper Jurassic, Karatau, Kazakhstan, 7. *Liadopsylla longiforceps* Becker-Migdisova, 1985—Upper Jurassic, Karatau, Kazakhstan, 8. *Liadopsylla tenuicornis* Martynov, 1926—Upper Jurassic, Karatau, Kazakhstan, 9. *Liadopsylla turkestanica* Becker-Migdisova, 1949—Upper Jurassic, Karatau, Kazakhstan, 10. *Gracilinervia mastimatoides* Becker-Migdisova, 1985—Upper Jurassic, Karatau, Kazakhstan, 11. *Malmopsylla karatavica* Becker- Migdisova, 1985 – Upper Jurassic, Karatau, Kazakhstan, 12. *Neopsylloides turutanovae* Becker-Migdisova, 1985—Upper Jurassic, Karatau, Kazakhstan, 13. *Pauropsylloides jurassica* Becker-Migdisova, 1985—Upper Jurassic, Karatau, Kazakhstan, 14. *Liadopsylla mongolica* Shcherbakov, 1988—Lower Cretaceous, Bon Tsagaan, Mongolia 15. *Liadopsylla apedetica* Ouvrard, Burckhardt et Azar, 2010—Lower Cretaceous, Lebanon, 16. *Liadopsylla lautereri* (Shcherbakov, 2020)—Lower Cretaceous, Buryatia, Russia 17. *Liadopsylla loginovae* (Shcherbakov, 2020)—Lower Cretaceous, Buryatia, Russia 18. *Stigmapsylla klimaszewskii* Shcherbakov, 2020—Lower Cretaceous, Buryatia, Russia 19. *Mirala burmanica* Burckhardt et Poinar, 2019—mid-Cretaceous, Kachin amber, 20. *Amecephala pusilla* gen. et sp. nov.—mid-Cretaceous, Kachin amber, 21. *Liadopsylla hesperia* Ouvrard et Burckhardt, 2010—Upper Cretaceous, Raritan amber, U.S.A.

Psyllids or jumping plant-lice are a group of small, generally host-specific plant-sap sucking insects with around 4000 described species^1^. A few species are major pests on fruits or vegetables, mostly by transmitting plant pathogens. Others damage forest plantations or ornamental plants by removal of plant-sap, stunting new growth, inducing galls or secreting honeydew and wax, an ideal substrate for sooty mould which reduces photosynthesis^2^_._ Modern psyllids, defined by the enlarged and immobile metacoxae in adults allowing them to jump, display a wide range of morphological diversity regarding the head, antennae, legs, forewings, terminalia, etc. in adults and body shape, antennal structure and the type of setae or wax pores in immatures. Modern psyllids are documented in the fossil record since the Eocene (Lutetian)^3^ (Fig. 1). The stem-group of modern psyllids constitutes, according to Burckhardt & Poinar, 2019^4^, the paraphyletic Liadopsyllidae Martynov, 1926^5^ with 17 species and six genera (*Liadopsylla* Handlirsch, 1925^6^, *Gracilinervia *Becker-Migdisova, 1985^7^, *Malmopsylla *Becker-Migdisova, 1985^7^, *Mirala *Burckhardt & Poinar, 2019^4^, *Neopsylloides* Becker-Migdisova, 1985^7^ and *Pauropsylloides* Becker-Migdisova, 1985^7^) from early Jurassic to late Cretaceous^4,8^. Shcherbakov^9^ added three species from the Lower Cretaceous for one of which he erected the genus *Stigmapsylla* and for the other two the subgenus *Liadopsylla* (*Basicella*). He also transferred two previously described species from *Liadopsylla* to *Cretapsylla* Shcherbakov^9^. Further he resurrected the Malmopsyllidae Becker-Migdisova, 1985^7^ splitting it into Malmopsyllinae (for *Gracilinervia*, *Malmopsylla*, *Neopsylloides* and *Pauropsylloides*) and Miralinae Shcherbakov^9^ (for *Mirala*). Apart from three species described from amber fossils, all Mesozoic psyllids are poorly preserved impression fossils of which usually only the forewing is preserved. The current classification of Mesozoic psyllids (Liadopsyllidae and Malmopsyllidae) is based almost exclusively upon forewing characters^7,9^, despite that several phylogenetically significant characters from other body parts have been described from amber inclusions^4,8^. Judging from the impression fossils, Liadopsyllidae and Malmopsyllidae appear morphologically quite homogeneous but this may be a result of the surprisingly scarce fossil record of psyllids compared to other insect groups. The discoveries of Cretaceous amber fossils radically alter this picture, e.g. the recently described *Mirala burmanica *Burckhardt & Poinar, 2019 from Myanmar amber^4^.

Here we describe a second taxon of Mesozoic psyllids from Kachin amber, *Amecephala pusilla* **gen. et sp. nov.**, possessing a series of characters unique within Mesozoic psyllids, discuss the phylogenetic relationships within the group, and provide an updated key to genera as well a checklist of recognised species (Table 1).

To satisfy a requirement by Article 8.5.3 of the International Code of Zoological Nomenclature this publication has been registered in ZooBank with the LSID: urn:lsid:zoobank.org:act:D3AF7597-47BF-4D6C-9020-982F4C20315E.

**Table 1:** Annotated checklist of known species of Liadopsyllidae Martynov, 1926^5^.

Taxon	Locality	Age and formation	Preservation	Sex
***Amecephala *gen. nov**				
*A. pusilla* **sp. nov.**	Myanmar, Kachin State, Hukawng Valley, SW of Maingkhwan, Noije Bum 2001 Summit Site amber mine	Mid-Cretaceous (Aptian/Cenomanian)	Amber inclusion	Female
***Gracilinervia *Becker-Migdisova, 1985**^7^**, p. 78**				
*G*. *mastigimatoides* Becker-Migdisova, 1985^7^, p.s 79	Kazakhstan, Karatau, Kasharata (Mikhailovka), Aulie	Upper Jurassic (Callovian); Karabastau Fm	Compression	Unknown
***Liadopsylla *Handlirsch, 1921**^6^**, p. 213**				
*L. apedetica* Ouvrard, Burckhardt et Azar, 2010 in Ouvrard et al. 2010^8^, p. 173	Lebanon, Mdeyrij-Hammana, Casa Baabda	Lower Cretaceous (late Barremian); Grès du Liban Fm	Amber inclusion	Female
*L. asiatica* Becker-Migdisova, 1985^7^, p. 74	Kazakhstan, Karatau, Kasharata (Mikhailovka), Aulie	Upper Jurassic (Callovian); Karabastau Fm	Compression	Unknown
*L. brevifurcata* Becker-Migdisova, 1985^7^, p. 69	Kazakhstan, Karatau, Kasharata (Mikhailovka), Aulie	Upper Jurassic (Callovian); Karabastau Fm	Impression	Unknown
*L. geinitzi* Handlirsch, 1921^6^, p. 213	Germany, Mecklenburg, Dobbertin	Lower Jurassic (early Toarcian); *Harpoceras falciferum* ammonoid zone	Impression	Unknown
*L. grandis* Becker-Migdisova, 1985^7^, p. 63	Kazakhstan, Karatau, Kasharata (Mikhailovka), Aulie	Upper Jurassic (Callovian); Karabastau Fm	Compression	Male
*L. hesperia* Ouvrard et Burckhardt, 2010 in Ouvrard et al. 2010^8^, p. 175	U.S.A., New Jersey, Middlesex County, Sayreville	Upper Cretaceous (Turonian); Raritan Fm	Amber inclusion	Female?
*L. karatavica* Becker-Migdisova, 1985^7^, p. 73	Kazakhstan, Karatau, Kasharata (Mikhailovka), Aulie	Upper Jurassic (Callovian); Karabastau Fm	Compression	Male
*L. lautereri* (Shcherbakov, 2020) ^9^, p. 130	Russia, SW Buryatia Zakamensk district, Khasurty, 10 km S of Tsakir	Lower Cretaceous (Aptian); Gusinoe Ozero Gr	Impression	Unknown
*L. loginovae* (Shcherbakov, 2020) ^9^, p. 132	Russia, SW Buryatia Zakamensk district, Khasurty, 10 km S of Tsakir	Lower Cretaceous (Aptian); Gusinoe Ozero Gr	Impression	Unknown
*L. longiforceps* (Becker-Migdisova, 1985)^7^, p. 61	Kazakhstan, Karatau, Kasharata (Mikhailovka), Aulie	Upper Jurassic (Callovian); Karabastau Fm	Compression	Unknown
*L. mongolica* Shcherbakov, 1988^13^, p. 61	Mongolia, Bon Tsagaan	Lower Cretaceous (Aptian); Dzun-Bain Fm., Khurilt Mb	Impression	Unknown
*L. obtusa* Ansorge, 1996^14^, p. 55	Germany, Mecklenburg-Vorpommern, Grimmen, Klein Lehmhagen pit	Lower Jurassic (early Toarcian); *Dactylioceras tenuicostatum* zone	Impression	Unknown
*L. tenuicornis* Martynov, 1926^5^, p. 1359	Kazakhstan, Karatau, Kasharata (Mikhailovka), Aulie	Upper Jurassic (Callovian); Karabastau Fm	Compression	Male
*L. turkestanica* Becker-Migdisova, 1949^15^, p. 42	Kazakhstan, Karatau, Kasharata (Mikhailovka), Aulie	Upper Jurassic (Callovian); Karabastau Fm	Compression	4 Males, 4 females
***Malmopsylla *Becker-Migdisova, 1985**^7^**, p. 76**				
*M. karatavica* Becker-Migdisova, 1985^7^: 76	Kazakhstan, Karatau, Kasharata (Mikhailovka), Aulie	Upper Jurassic (Callovian); Karabastau Fm	Impression	Unknown (forewing only)
***Mirala *Burckhardt et Poinar, 2019**^4^**, p. 3**				
*M. burmanica* Burckhardt et Poinar, 2019^4^, p. 3	Myanmar, Kachin State, Hukawng Valley, SW of Maingkhwan, Noije Bum 2001 Summit Site amber mine	Mid-Cretaceous (Aptian/Cenomanian)	Amber inclusion	Unknown
***Neopsylloides *Becker-Migdisova, 1985**^7^**, p. 77**				
*N. turutanovae* Becker-Migdisova, 1985^7^, p. 77	Kazakhstan, Karatau, Kasharata (Mikhailovka), Aulie	Upper Jurassic (Callovian); Karabastau Fm	Compression	Unknown
***Pauropsylloides *Becker-Migdisova, 1985**^7^**, p. 79**				
*P. jurassica* Becker-Migdisova, 1985^7^, p. 79	Kazakhstan, Karatau, Kasharata (Mikhailovka), Aulie	Upper Jurassic (Callovian); Karabastau Fm	Compression	Unknown
***Stigmapsylla *Shcherbakov, 2020**^9^**, p. 129**				
*S. klimaszewskii* Shcherbakov, 2020^9^: 130	Russia, SW Buryatia Zakamensk district, Khasurty, 10 km S of Tsakir	Lower Cretaceous (Aptian); Gusinoe Ozero Gr	Impression	Unknown

All known specimens are adults. Becker-Migdisova (1985, p. 62)^7^ synonymised *Asientomum* Martynov, 1926^5^, p. 1364 (replacement name for *Lithentomum* Martynov, 1926^5^, p. 1365, *nec* Scudder, 1867^16^, p. 206) with *Liadopsylla* and transferred *Lithentomum praecox* Martynov, 1926^5^, p. 1365, to *Liadopsylla*. The venation of the forewing and particularly of the hindwing as described by Martynov^5^ clearly places this species in the Psocodea. Pending an examination of the holotype we follow Martynov^5^ rather than Becker-Migdisova^7^ in this matter.

### Systematic palaeontology

Order Hemiptera Linnaeus, 1758^17^

Suborder Sternorrhyncha Amyot et Audinet-Serville, 1843^18^

Superfamily Psylloidea Latreille, 1807^19^

Family Liadopsyllidae Martynov, 1926^5^

### Genus †*Amecephala* gen. nov

urn:lsid:zoobank.org:act:9DABC236-FFB9-4305-82EC-4E293212849B

#### Type species

† *Amecephala pusilla* sp. nov., by present designation and monotypy.

*Etymology* From ancient Greek ἡ άμε [ē áme] = shovel and ἡ κεφαλή [ē kefalé] = head for its shovel-shaped head. Gender: feminine.

#### Diagnosis

**Figure 2:**
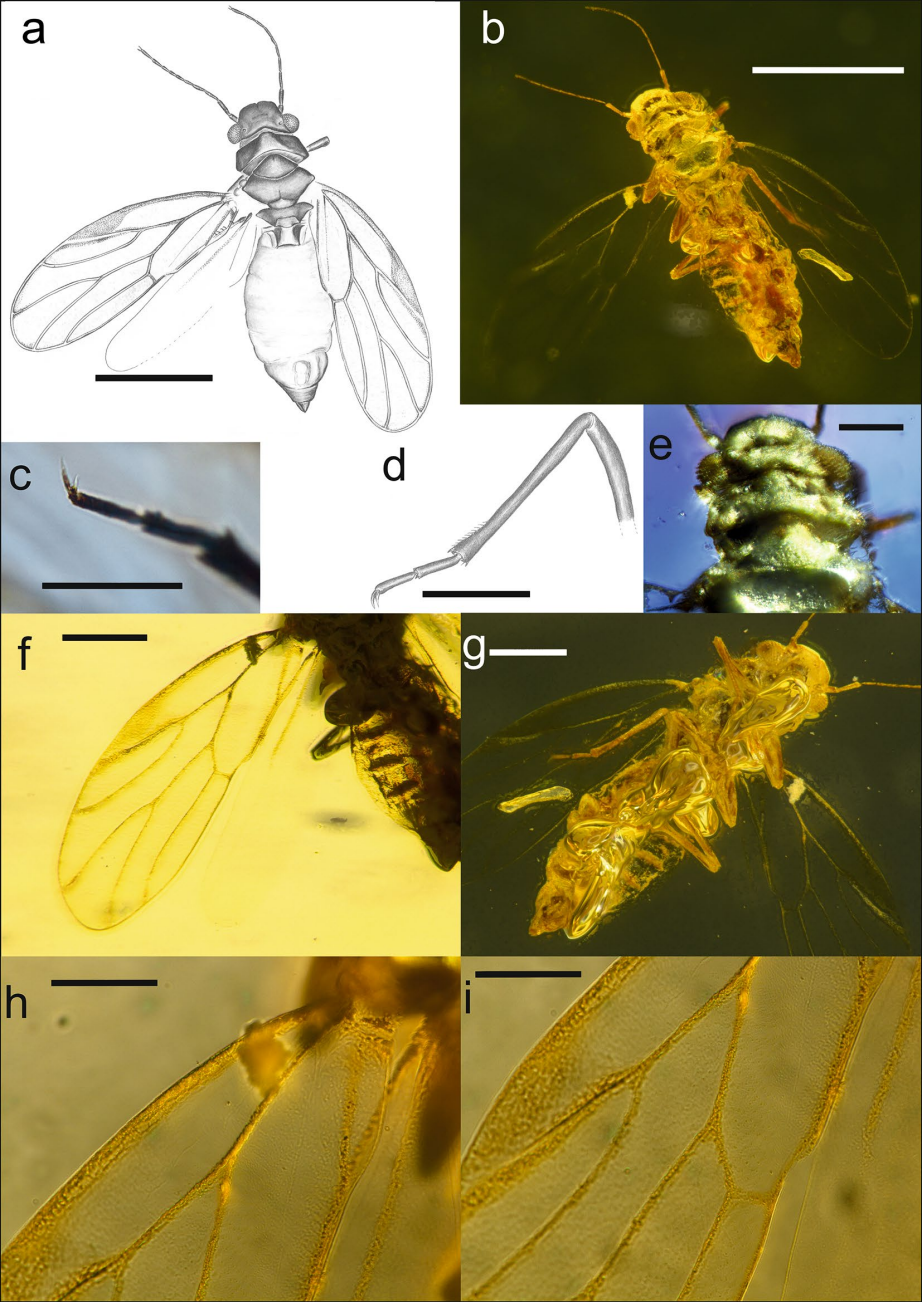
(**a**‒**i**) *Amecephala pusilla* **gen. et sp. nov.** imago. Drawing of body in dorsal view (**a**), Body in dorsal view (**b**), Metatarsus (**c**), Drawing of hind leg (**d**), Head in dorsal view (**e**), Forewing (**f**), Body in ventral view (**g**), Basal part of claval suture (**h**), Distal part of claval suture (**i**); Scale bars: 0.5 mm (**a**,**b**); 0.2 mm (**f**,**g**); 0.1 mm (**c**,**d**,**e**,**h**,**i**).

Vertex rectangular; coronal suture developed in apical half; median ocellus on ventral side of head, situated at the apex of frons which is large, triangular; genae not produced into processes; toruli oval, medium sized, situated in front of eyes below vertex. Eyes hemispheric, relatively small (Fig. 2a,b,e,g). Antenna with pedicel about as long as flagellar segments 1 and 8, longer than remainder of segments. Pronotum ribbon-shaped, relatively long, laterally of equal length as medially. Forewing (Fig. 2a,b,f,g) elongate, widest in the middle, narrowly rounded at apex; pterostigma short and broad, triangular, not delimited at base by a vein thus vein R_1_ not developed; veins R and M + Cu subequal in length; vein Rs relatively short, slightly curved towards fore margin; vein M shorter than its branches which are of subequal length; cell cu_1_ low and very long. Female terminalia short, cuneate.

#### Description

Head weakly inclined from longitudinal body axis; about as wide as pronotum and mesoscutum, dorso-ventrally compressed. Vertex rectangular; anterior margin weakly curved, indented in the middle; posterior margin slightly concavely curved; coronal suture developed in apical half, basal half not visible; lateral ocelli near posterior angles of vertex, hardly raised; median ocellus on ventral side of head, situated at the apex of frons which is large, triangular; genae not produced into processes; preocular sclerites lacking; toruli oval, medium sized, situated in front of eyes below vertex; clypeus partly covered by gas bubble, appearing flattened, pear-shaped. Eyes hemispheric, relatively small (Fig. 2a,b,e,g). Antenna 10-segmented, filiform, moderately long, flagellum 1.6 times as long as head width; pedicel very long, about as long as flagellar segments 1 and 8; rhinaria not visible (Fig. 2a,b). Thorax (ventrally not visible) with pronotum wider than mesopraescutum as wide as mesoscutum, laterally of the same length as medially. Mesothorax large; mesopraescutum triangular, with arcuate anterior margin, almost twice wider than long in the middle; mesopraescutum slightly longer than pronotum in the middle; mesoscutum subtrapezoid with slightly arched anterior margin, about 3.0 times wider than long in the middle; delimitation between mesoscutum and mesoscutellum clearly visible. Metascutellum trapezoid, narrower than mesoscutellum with a submedian longitudinal low ridge on either side. Parapterum and tegula forming small oval structures of about the same size; the former slightly in front of the latter. Forewing (Fig. 2a,b,f) membranous, elongate, narrow at base, widest in the middle, narrowly rounded at apex which lies in cell m_1_ near the apex of vein M_3+4_; vein C + Sc narrow; cell c + sc long, widening toward apex; costal break not visible, perhaps absent; pterostigma short and broad, triangular, not delimited at base by a vein thus vein R_1_ not developed; vein R + M + Cu relatively short; veins R and M + Cu subequal in length; vein R_2_ relatively short and straight; vein Rs relatively short, slightly curved towards fore margin; vein M shorter than its branches which are of subequal length; vein Cu short, splitting into very long Cu_1a_ and short Cu_1b_, hence cell cu_1_ low and very long; claval suture visible (Fig. 2h,i); anal break near to apex of vein Cu_1b_ (Fig. 2f,i). Hindwing (Fig. 2a) shorter than forewing, more than twice as long as wide, membranous; venation indistinct. Legs similar in shape and size, long, slender (Fig. 2c,d,g); femora slightly enlarged distally, tibiae long and slightly enlarged distally; metatibia lacking genual spine and apical sclerotized spurs, but bearing several apical bristles and, in distal quarter, a row of short bristles (Fig. 2d); tarsi two-segmented, tubular of similar length though basal segment slightly thicker than apical one, claws large, one-segmented, pulvilli absent (Fig. 2c–d). Abdomen appearing flattened, tergites and sternites not clearly visible. Female terminalia short, slightly shorter than head width, cuneate (Fig. 2a,b,g).

### Revised key to Mesozoic psylloid genera (after Burckhardt & Poinar^4^, modified)

1. Forewing lacking pterostigma…...........................................................................................................................*Liadopsylla* Handlirsch, 1921 (= *Cretapsylla* Shcherbakov, 2020 **syn. nov.**; = *Basicella* Shcherbakov, 2020 **syn. nov.**)
  -Forewing bearing pterostigma…...................................................................................................................................................................................................................................................................................................................2
2. Vein Rs in forewing straight, veins Rs and M subparallel; vein M not branched; vein R shorter than M + Cu; vein Cu_1b_ almost straight, directed toward wing base…*.......................................................Mirala* Burckhardt et Poinar, 2020
  -Combination of characters different. Vein Rs in forewing concavely curved towards fore margin (not visible in *Stigmapsylla*), veins Rs and M from base to apex first converging then diverging; vein M branched; vein Cu_1b_ straight or curved, directed toward hind margin or apex of wing…....................................................................................................................................................................................................................................................................................3
3. Vein R of forewing distinctly shorter than M + Cu…........................................................................................................................................................................................................................................*Stigmapsylla* Shcherbakov, 2020
  -Vein R of forewing distinctly longer than M + Cu, or veins R and M + Cu subequal in length…...................................................................................................................................................................................................................4
4. Vein R of forewing distinctly longer than M + Cu; vein Cu_1a_ almost straight…*......................................................................................................................................................................................Malmopsylla* Becker-Migdisova, 1985
  -Veins R and M + Cu of forewing subequal in length; vein Cu_1a_ distinctly curved…......................................................................................................................................................................................................................................5
5. Forewing with cell cu_1_ low and very long, around 6.0 times as long high…....................................................................................................................................................................................................................*Amecephala* **gen. nov.**
  -Forewing with cell cu_1_ higher and shorter, less than 2.5 times as long high…..............................................................................................................................................................................................................................................6
6. Forewing with long pterostigma, vein R_2_ straight…*.............................................................................................................................................................................................................................Neopsylloides* Becker-Migdisova, 1985
  -Forewing with short pterostigma, vein R_2_ curved….....................................................................................................................................................................................................................................................................................7
7. Vein R + M + Cu of forewing ending at basal quarter of wing…...........................................................................................................................................................................................................*Gracilinervia* Becker-Migdisova, 1985
  -Vein R + M + Cu of forewing ending at basal third of wing…*........................................................................................................................................................................................................Pauropsylloides* Becker-Migdisova, 1985

### †*Amecephala pusilla* sp. nov

urn:lsid:zoobank.org:act:6B20A4F4-57DB-4F06-A43C-5DE3653D76E3 (Fig. 2a–i)

#### Etymology

From Latin pusillus = tiny, very small—for its small body size.

#### Holotype

Female, specimen number MAIG 6686; deposited in the Museum of Amber Inclusion, University of Gdańsk, Gdańsk, Poland. Complete and well-preserved (Fig. 2b,g), probably slightly compressed dorso-ventrally; the wings appear slightly detached from thorax and have been probably forced away from the thorax by the compression. Several gas bubbles on the ventral body side obscure parts of the head, thorax, abdomen, legs and the right forewing (Fig. 2g). Syniclusions: Aleyrodidae (part; second part in broken piece).

#### Locality and stratum

Myanmar, Kachin State, Hukawng Valley, SW of Maingkhwan, former Noije Bum 2001 Summit Site amber mine (closed). Lowermost Cenomanian, Upper Cretaceous.

#### Species diagnosis

As for the genus.

#### Description

Female; male unknown. Body minute, 1.20 mm long including forewing when folded over body. Head (ventrally partly covered by gas bubble) 0.28 mm wide, 0.10 mm long; vertex width 0.20 mm wide, 0.09 mm long; microsculpture or setae not visible. Antenna (Fig. 2a,b) with globular scape and cylindrical pedicel, thinner and longer than scape; flagellum 0.40 mm long; 1.6 times as long as head width; flagellar segments slightly more slender than pedicel, relative lengths as 1.0:0.7:0.6:0.6:0.6:0.6:0.7:1.0; flagellar segment 8 bearing two subequal terminal setae shorter that the segment. Clypeus and rostrum not visible, covered by gas bubble. Forewing (Fig. 2a,b,f,g) 0.90 mm long, 0.30 mm wide, 3.0 times as long as wide; membrane transparent, colourless, veins pale; anterior margin curved basally, posterior margin almost straight; vein R + M + Cu ending in basal fifth of wing; vein R slightly shorter that M + Cu; bifurcation of vein R proximal to middle of wing; cell r_1_ relatively narrow; vein R_2_ distinctly shorter than Rs; vein Rs relatively short, strongly curved towards fore margin; vein M slightly longer than veins R and M + Cu; M branching proximal to Rs–Cu_1a_ line; cell m_1_ value more than 2.6, cell cu_1_ value more than 6.0; surface spinules not visible. Hindwing (Fig. 2b,f) membranous, transparent and colourless. Female terminalia (Fig. 2a,b,g) with apically pointed proctiger; circumanal ring irregularly oval, about half as long as proctiger.

## Discussion

Recent molecular phylogenetic analyses of modern Psylloidea^20^ support largely the classification by Burckhardt & Ouvrard^1^ which is based to a great extent on the morphology of immatures (see also White & Hodkinson^21^) but also on adult characters such as details of the head, legs and terminalia. The venation of the forewing is rarely diagnostic for taxa at or above generic rank due to the high degree of homoplasy^1,6^. A good example is the presence or absence of a pterostigma, though stable in most genera it varies sometimes, as in *Gyropsylla* Brèthes, 1921^22^. Shcherbakov^9^ resurrected the Malmopsyllidae, synonymised with Liadopsyllidae by Burckhardt & Poinar^4^, and split it into the two subfamilies Malmopsyllinae and Miralinae using evidence from eight forewing characters (Table 2). A critical review of these characters including in modern Psylloidea shows that they are unsuitable for diagnosing families and subfamilies. Five of the characters are variable within modern genera and one character is poorly defined (Cu [CuA] fork: not clear if it refers to the angle or the shape of cell Cu_2_). The remaining two characters constitute autapomorphies defining *Mirala* but leaving Liadopsyllidae sensu Shcherbakov^9^, Malmopsyllidae sensu Shcherbakov^9^ and Malmopsyllinae sensu Shcherbakov^9^ undefined (plesiomorphies!) in a phylogeny based classification^23^. They are, therefore rejected here. We propose following formal synonymies: Liadopsyllidae Martynov, 1927^5^; = Malmopsyllidae Becker-Migdisova, 1985^7^, **stat. rev.**; = Miralinae Shcherbakov, 2020^9^, **syn. nov.**

**Table 2:** Forewing characters used by Shcherbakov^9^ to define the families and subfamilies.

Character	Liadopsyllidae	Malmopsyllidae	Malmopsyllinae	Miralinae	Modern Psylloidea (NHMB data)	Polarity, apomorphic state
Costal space	Elongate, usually ribbon-shaped	Widening proximally or distally	Not widening distally	Widening distally	Variable, sometimes within a genus	Unknown
Pterostigma	Usually present, but often poorly developed	Distinct, dark	Not mentioned	Not mentioned	Variable, sometimes within a genus	Unknown
R + M + Cu [R + M + CuA] bifurcation	at < 1/5 wing length	at > 1/4 wing length	at 1/4–1/3 wing length	before wing midlength	variable, sometimes within a genus	unknown
R bifurcation	At < 1/3 wing Length	At > 0.4 wing length	At 0.4–0.5 wing length	Beyond wing midlength	Variable, often within a genus	Unknown
M	Forked	Not mentioned	Forked	Unforked	Usually unforked	Unforked
M + Cu [M + CuA] bifurcation	Not mentioned	Not mentioned	At 0.35–0.5 wing length	Beyond wing midlength	Variable, sometimes within a genus, intergrading	Unknown
Cu [CuA] fork	Triangular	Not mentioned	Triangular	Broad subquadrangular	Character poorly defined	Unknown
Cu_1b_ [CuA2]	Not recurrent or very short	Rather long, sometimes recurrent	Long, not recurrent	Recurrent	Variable	Unknown

Nomenclature of veins follows Burckhardt & Poinar^4^, terms used by Shcherbakov^9^ are given in brackets.

Similarly problematical are the circumscriptions of *Cretapsylla* Shcherbakov, 2020^9^, *Liadopsylla* (subgenus *Basicella* Shcherbakov, 2020^9^) and *Stigmapsylla* Shcherbakov, 2020^9^. The first is separated from *Liadopsylla* by the length ratio of the veins M + Cu and Cu (> 4 versus < 2) and the stronger curved vein M. Both characters vary within genera in modern psyllids and are unsuitable for defining genera. Shcherbakov^9^ provides a putative autapomorphy (“free CuA base”) for the monophyly of the subgenus *Basicella* but fails to document the monophyly of the subgenus *Liadopsylla *sensu Shcherbakov^9^. For these reasons we propose following synonymies: *Liadopsylla* Handlirsch, 1921^6^; = *Cretapsylla* Shcherbakov, 2020^9^, **syn. nov.**; = *Basicella* Shcherbakov, 2020^9^), **syn. nov.** and following revised combinations: *Liadopsylla apedetica* Ouvrard, Burckhardt et Azar, 2010^8^, **comb. rev.** and *Liadopsylla hesperia* Ouvrard et Burckhardt, 2010^8^, **comb. rev.** both from *Cretapsylla* Shcherbakov, 2020^9^. The monotypic *Stigmapsylla* Shcherbakov, 2020^9^ is represented by a single, incomplete forewing and represents yet another other poorly defined liadopsyllid genus (along with *Gracilinervia*, *Malmopsylla*, *Neopsylloides and Pauropsylloides*).

*Amecephala pusilla* **gen. et sp. nov.** differs from the other known taxa of Liadopsyllidae in the very long pedicel of the antenna, the long and narrow forewings (3.0 times as long as wide), that are widest in the middle, the very short vein Rs as well as the very long and low cell cu_1_. It shares with *Liadopsylla* the absence of vein R_1_ and the short vein R + M + Cu ending at basal fifth of wing. Whether these characters reflect a close phylogenetic relationship is difficult to judge as these characters are strongly subjected to homoplasy. Unlike *Liadopsylla*, *Amecephala* displays a distinctly pigmented pterostigma as the other Mesozoic Liadopsyllidae.

The antenna of *Amecephala pusilla* shows some remarkable features. In Psylloidea, including Liadopsyllidae, the scape and, to a lesser extent, the pedicel, are in general distinctly wider but much shorter than any of the flagellar segments. In most psyllids, one of the antennal segments 3, 7 or 8 (flagellar segments 1, 5 or 6) constitutes the longest segment. There are a few exceptions such as *Livia* Latreille, 1802^24^, *Notophyllura* Hodkinson, 1986^25^, or some species of *Calophya* Löw, 1879^26^, where the pedicel is longer than the other segments. These taxa have short antennae (usually shorter than head width) and sometimes a reduced number of antennal segments. In *Amecephala pusilla*, the antenna is distinctly longer than the head width and scape and pedicel are almost as slender as the flagellar segments. The long pedicel is an unique feature in Liadopsyllidae and very exceptional in modern psyllids and constitutes probably an apomorphic condition which developed apparently several times independently, in modern psyllids mostly by reduction of the flagellar length. The general head shape of *Amecephala* is similar to that of *Liadopsylla* and *Mirala*; the compound eyes in *Liadopsylla* are less protruding than in the other two genera.

The legs of *Amecephala*, *Liadopsylla* and *Mirala* are of similar build. The hind legs are not modified compared to those in modern psyllids, the tarsal segments are subequal in length and lack pulvilli. Whereas the first two characters are primitive, the last one is derived. Pulvilli or similar structures are present in adults of modern psyllids, in whiteflies, aphids, male scale insects and several groups of Auchenorrhyncha and Heteroptera^27^. The reduction of pulvilli in Liadopsyllidae constitutes a potential autapomorphy supporting, admittedly weakly, the monophyly of Liadopsyllidae.

Little is known about the terminalia of Liadopsyllidae. In modern psyllids, the terminalia constitute often the most important structure to diagnose species. The male terminalia of following species have been described: *Liadopsylla grandis* Becker-Migdisova, 1985^7^, *Liadopsylla karatavica* Becker-Migdisova, 1985^7^, *Liadopsylla longiforceps* (Becker-Migdisova, 1985)^7^, *Liadopsylla tenuicornis* Martynov, 1926^5^, and *Liadopsylla turkestanica* Becker-Migdisova, 1949^15^. Of the last species, also the female terminalia have been described. All these species are represented by compression fossils, sometimes difficult to interpret and lacking morphological detail. More details are visible in the amber specimen of *Liadopsylla apedetica* Ouvrard, Burckhardt et Azar, 2010^8^, a female displaying very long terminalia. The female terminalia of *L. turkestanica* appear much shorter. In *Amecephala pusilla* the female terminalia are relatively short and an oval circumanal ring is visible. This structure, always present in modern psyllids^28^, is documented here for the first time in Mesozoic psyllids. In modern psyllids, the length of the female terminalia is often correlated with the place where the eggs are laid. Short female terminalia are usually present in species that lay their eggs on the surface or in crevices of a twig or at the base of leaf or flower buds, as in many species of *Cacopsylla* Ossiannilsson, 1970^29^. Long terminalia are used for depositing the eggs into buds, such as in the Holarctic species of *Psylla* Geoffroy, 1762^30^, associated with Betulaceae, or into the flower heads of Asteraceae as in species of the predominantly Neotropical *Calinda* Blanchard, 1852^31^, Burckhardt, pers. obs. This diversity of female terminalia in Liadopsyllidae suggests that the family may have used a range of substrates for oviposition perhaps on different host taxa. According to Burckhardt & Poinar^4^ the Lauraceae could have been among the host families of psyllids from Burmese amber.

## Material and methods

The specimen is an inclusion in mid-Cretaceous amber from the Kachin State in northern Myanmar (Burma). The specimen was purchased together with the whole bunch in 2016 from authorised dealer in Bahan, registered by Ministry of Co-operatives in Myanmar. To further prove sample origination, VIS and UV (395 nm) examination of sample was proceeded at Laboratory of Amber, Museum of Amber Inclusions, University of Gdańsk and Fourier Transform Infrared Spectrum with use of Nicolet iS10 in Amber Laboratory of the International Amber Association in Gdańsk. The amber piece was cut and polished for better visibility. For the microscopic examination, we used a Nikon SMZ1500, Nikon SMZ1270, Leica M205C stereoscopic microscopes and a Nikon Microphot-FX equipped with a camera lucida and changeable direct and transmitted light. The photographs were taken using a Nikon Microphot-FX with a Nikon Eclipse E 600 digital camera and Lucia software and edited with Adobe Photoshop Elements 6.0.

Morphological terminology follows mostly Ossiannilsson^28^ and Hollis^32^ but the interpretation of veins R_1_ and R_2_ accords with Becker-Migdisova^5^ and Burckhardt & Poinar^6^.

## References

[1] Burckhardt D, Ouvrard D (2012). A revised classification of the jumping plant-lice (Hemiptera: Psylloidea). Zootaxa.

[2] Burckhardt D (1994). Psylloid pests of temperate and subtropical crop and ornamental plants (Hemiptera, Psylloidea): A review. Trends Agric. Sci. Entomol..

[3] Ouvrard D, Burckhardt D, Greenwalt D (2013). The oldest jumping plant-louse (Hemiptera: Sternorrhyncha) with comments on the classification and nomenclature of the Palaeogene Psylloidea. Acta Mus. Morav. Sci. Biol..

[4] Burckhardt D, Poinar G (2019). The first jumping plant-louse from mid-Cretaceous Burmese amber and its impact on the classification of Mesozoic psylloids (Hemiptera: Sternorrhyncha: Psylloidea s.l.). Cret. Res..

[5] Martynov AV (1926). Jurassic fossil insects from Turkestan. 6. Homoptera and Psocoptera. Bull. Acad. Sci. U.R.S.S..

[6] Handlirsch A, Schrӧder C (1921). Palӓontologie. Handbuch der Entomologie. Bd. III.

[7] Becker-Migdisova EE (1985). Iskopaemye nasekomye psillomorfy (Fossil psyllomorphous insects). Trudy Paleontol. Inst..

[8] Ouvrard D, Burckhardt D, Azar D, Grimaldi D (2010). Non-jumping plant-lice in Cretaceous amber (Hemiptera: Sternorrhyncha: Psylloidea). Syst. Entomol..

[9] Shcherbakov DE (2020). New Homoptera from the early Cretaceous of Buryatia with notes on the insect fauna of Khasurty. Russ. Entomol. J..

[10] Drohojowska J, Szwedo J (2015). Early Cretaceous Aleyrodidae (Hemiptera: Sternorrhyncha) from the Lebanese amber. Cret. Res..

[11] Hakim M, Azar D, Szwedo J, Brysz AM, Huang DY (2019). New paraneopterans (Protopsyllidioidea, Hemiptera) from the mid-Cretaceous amber of northern Myanmar. Cret. Res..

[12] Drohojowska J, Szwedo J, Żyła D, Huang D-Y, Müller P (2020). Fossils reshape the Sternorrhyncha evolutionary tree (Insecta, Hemiptera). Sci. Rep..

[13] Shcherbakov DE (1988). Novye mezozoïskie ravnokrylye (New Mesozoic Homoptera.) in Novye vidy iskopaemykh bespozvonochnykh Mongolii (New species of fossil invertebrates of Mongolia) (ed. Rozanov, A. Yu.). Sov.-Mongol. Paleontol. Exped..

[14] Ansorge J (1996). Insekten aus dem oberen Lias von Grimmen (Vorpommern, Norddeutschland). Neue Paläontol. Abhandl..

[15] Becker-Migdisova EE (1949). Mezozoiskie Homoptera Srednei Azii (Mesozoic Homoptera of Central Asia). Trudy Paleontol. Inst..

[16] Scudder SH (1867). Taxonomic names. On some remains of Palaeozoic insects recently discovered in Nova Scotia and New Brunswick. Can. Nat..

[17] Linnaeus, C. *Systema naturae per regna tria naturae, secundum classes, ordines, genera, species, cum characteribus, differentiis, synonymis, locis. Tomus I. Editio decima, reformata*. (Laurentius Salvius, Holmia, 1758).

[18] Amyot CJ-B, Audinet-Serville JG (1843). Deuxième partie. Homoptères. Homoptera Latr. Histoire naturelle des insects. Hemiptères.

[19] Latreille PA (1807). *Genera crustaceorum* et insectorum secundum ordinem naturalem in familias disposita, iconibus exemplisque plurimis explicate.

[20] Percy DM (2018). Resolving the psyllid tree of life: Phylogenomic analyses of the superfamily Psylloidea (Hemiptera). Syst. Entomol..

[21] White IM, Hodkinson ID (1985). Nymphal taxonomy and systematics of the Psylloidea (Homoptera). Bull. Brit. Mus. Nat. Hist. Entomol..

[22] Burckhardt D, Queiroz DL (2013). Systematics of the Neotropical jumping plant-louse genus *Limataphalara* (Hemiptera: Psylloidea: Aphalaridae) and phylogenetic relationships within the subfamily Aphalarinae. In *Studies in Hemiptera in honour of Pavel Lauterer and Jaroslav L. Stehlík* (eds Kment, P., Malenovský, I. & Kolibác, J.). *Acta Mus. Moraviae*. Sci. Biol..

[23] Hennig W (1950). Grundzüge einer Theorie der Phylogenetischen Systematik.

[24] Latreille PA (1802). Histoire naturelle, générale et particulière des crustacés et des insectes: ouvrage faisant suite aux oeuvres de Leclerc de Buffon, et partie du cours complet d’histoire naturelle rédigé par C.S. Sonnini.

[25] Hodkinson ID (1986). First records of Euphyllurini (Homoptera: Psylloidea) from Central and South America. Entomol. Scand..

[26] Löw F (1879). Zur Systematik der Psylloden. Verh. d. k. k. Zool.-Bot. Ges..

[27] Beutel RG, Friedrich F, Ge S-Q, Yang X-K (2014). Insect Morphology and Phylogeny.

[28] Ossiannilsson F (1992). The Psylloidea (Homoptera) of Fennoscandia and Denmark. Fauna Entomol. Scand..

[29] Ossiannilsson F (1978). Contributions to the knowledge of Swedish psyllids (Hem. Psylloidea). Entomol. Scand..

[30] Geoffroy EL (1762). Histoire abrégée des insectes qui se trouvent aux environs de Paris; dans laquelle ces animaux sont rangés suivant un ordre méthodique.

[31] Blanchard E, Gay C (1852). Orden VII. Hemípteros; V. Afidídeos; Tribu I.—Silinas. Historia física y política de Chile. Zoología.

[32] Hollis D (2004). Australian Psylloidea: Jumping plant lice and lerp insects.

